# Translation, Cultural Adaptation, and Psychometric Validation of the McMaster Family Assessment Device’s General Functioning Subscale: A Cross‐Sectional Study Among African Adults With Type 2 Diabetes

**DOI:** 10.1155/jdr/7959903

**Published:** 2026-04-28

**Authors:** Halimatou Alaofè, Raymond Yurika, Abidemi Okechukwu, Waliou Amoussa Hounkpatin, Lionel Medenou, John Ehiri

**Affiliations:** ^1^ The University of Arizona Mel and Enid Zuckerman College of Public Health, Tucson, Arizona, USA; ^2^ Université d’Abomey-Calavi Faculté des Sciences Agronomiques Cotonou, Littoral Department, Cotonou, Benin; ^3^ Université d’Abomey-Calavi, Faculté des Sciences de la Santé, Cotonou, Littoral Department, Benin, uac.bj

**Keywords:** FAD-GF12, family functioning, Francophone Africa, glycemic control, psychometric validation, type 2 diabetes

## Abstract

**Background:**

Family functioning plays a critical role in diabetes self‐management and glycemic control, yet validated tools for assessing family dynamics in Francophone African contexts remain limited. This study aimed to translate, culturally adapt, and psychometrically validate the French version of the Family Assessment Device—General Functioning Subscale (FAD‐GF12) among adults with type 2 diabetes (T2D) in Benin.

**Methods:**

A cross‐sectional psychometric validation study was conducted among 512 adults with T2D recruited from six healthcare centers. Translation and cultural adaptation followed established guidelines, incorporating expert review and participant feedback. The sample was randomly split for exploratory factor analysis (EFA; *n* = 256) and confirmatory factor analysis (CFA; *n* = 256). Reliability was assessed using Cronbach’s alpha and intraclass correlation coefficients (ICCs). Construct validity was examined using linear regression (continuous hemoglobin A1C–HbA1C) and logistic regression (HbA1c ≤7%) models.

**Results:**

The French FAD‐GF12 demonstrated good internal consistency (*α* = 0.85) and satisfactory test–retest reliability (ICC > 0.75). EFA and CFA supported a two‐factor structure distinguishing positive and negative family functioning, with good model fit (comparative fit index [CFI] = 0.94, Tucker–Lewis index [TLI] = 0.92, root mean square error of approximation [RMSEA] = 0.06, standardized root mean square residual [SRMR] = 0.04). The two factors were moderately correlated (*r* = 0.58), indicating related but distinct constructs. Better family functioning was significantly associated with lower HbA1c levels (*β* = −0.35, *p* = 0.003) and higher odds of good glycemic control (AOR = 2.04; 95% CI: 1.27–3.27; *p* = 0.004).

**Conclusions:**

The French FAD‐GF12 is a reliable, valid, and culturally appropriate tool for assessing family functioning among adults with T2D in Benin. Its demonstrated association with glycemic control highlights the importance of integrating family‐centered approaches into diabetes care in Francophone African settings.

## 1. Background

Type 2 diabetes (T2D) is a major global health concern, affecting an estimated 537 million adults worldwide and imposing substantial personal, social, and economic burdens [1–3]. Effective management requires sustained lifestyle modifications, including dietary regulation, physical activity, and medication adherence, behaviors strongly influenced by family and social environments [2, 4–8].

In African contexts, family systems play a central role in health and well‐being. Immediate and extended family networks provide emotional, informational, and practical support that can facilitate adherence and improve health outcomes [9–11]. Positive family functioning has been associated with better self‐management and glycemic control, whereas dysfunctional dynamics contribute to poor adherence and adverse metabolic outcomes [12–14]. Although family‐centered approaches are increasingly recognized, evidence from low‐ and middle‐income African settings remains limited [15, 16].

Despite the importance of family dynamics, appropriate tools for assessing family functioning in African populations are limited. Existing instruments, such as the Family Adaptability and Cohesion Evaluation Scales (FACES), the Family Environment Scale (FES), and the family attachment and changeability index (FACI8), often focus on specific relational dimensions, are relatively complex, and have been primarily validated in Western or non‐Francophone populations [17, 18]. Their applicability in Francophone African settings, characterized by extended family structures, collective responsibility, and distinct sociocultural norms, remains uncertain [9, 10, 19–21].

The McMaster Family Assessment Device (FAD) provides a theoretically grounded and multidimensional framework for assessing family functioning. Based on the McMaster Model, it evaluates key relational processes, including communication, problem‐solving, roles, and affective involvement [22–24]. These domains align closely with collectivist family systems common in African contexts, where interdependence and shared decision‐making are central. Its 12‐item General Functioning Subscale (FAD‐GF12) offers a concise yet comprehensive measure of overall family functioning, making it well suited for resource‐constrained settings [25, 26]. Unlike FACES and FES, it captures global family functioning while remaining sensitive to both supportive and dysfunctional interactions. The FAD‐GF12 has also been widely applied in studies of chronic illness, including diabetes and mental health, where family dynamics influence adherence, psychological well‐being, and clinical outcomes [18, 27–31]. Prior validations across diverse populations (e.g., Portuguese, Spanish, Dutch, and Malaysian) further support its reliability and cross‐cultural applicability [28, 32–35].

However, despite this evidence, the FAD‐GF12 has not been rigorously validated in Francophone African populations. Given the linguistic diversity and cultural specificity of these settings, careful translation and cultural adaptation are necessary to ensure validity. Therefore, this study aims to translate, culturally adapt, and validate the FAD‐GF12 for French‐speaking adults with T2D in the Republic of Benin and to examine its association with glycemic control. We hypothesize that healthier family functioning will be associated with better glycemic control. A culturally appropriate and validated tool will support the integration of family‐centered approaches into diabetes care and improve health outcomes in Francophone African populations.

## 2. Methods

### 2.1. Study Design and Setting

We conducted a cross‐sectional psychometric validation study between October and November 2023 in Parakou and Cotonou, Republic of Benin, in accordance with the STROBE (Strengthening the Reporting of Observational Studies in Epidemiology) guidelines. The study was implemented in collaboration with six secondary healthcare centers providing outpatient care for adults with T2D. The primary objective was to translate, culturally adapt, and psychometrically validate the French version of the FAD‐GF12 instrument.

### 2.2. Instrument Description

The FAD‐GF12 comprises 12 items assessing six domains of family functioning: problem‐solving, communication, roles, affective responsiveness, affective involvement, and behavioral control [25, 26]. Each item is rated on a 4‐point Likert scale, with six positive and six negative worded items. Lower mean scores indicate healthier family functioning.

### 2.3. Translation and Cultural Adaptation

The translation process followed established cross‐cultural adaptation guidelines [36–38]. A dual‐panel, concept‐based approach ensured linguistic accuracy and conceptual equivalence. A conceptual review panel, comprising two bilingual teachers and three healthcare professionals (a nurse, a public health specialist, and a nutritionist), reviewed and harmonized the translations to preserve conceptual consistency with the original English instrument. A language review panel, consisting of five monolingual French speakers (one nurse, one teacher, and three adults with T2D), evaluated item clarity, naturalness, and cultural relevance. Minor linguistic and contextual revisions were made to improve comprehension and ensure cultural alignment prior to pretesting.

### 2.4. Pretesting and Content Validation

A mixed‐methods exploratory sequential design was employed to assess the face and content validity of the French FAD‐GF12 [39]. In the first phase, structured interviews were conducted with an expert panel of eight bilingual professionals (two nurses, two public health specialists, two pharmacists, and two teachers). These interviews evaluated item clarity, comprehension, semantic equivalence, and cultural appropriateness. Feedback from the expert panel led to minor revisions in wording and phrasing to improve clarity and contextual alignment [40].

In the second phase, a focus group discussion (FGD) was conducted with six adults with T2D who met the inclusion criteria (≥40 years, diagnosed for ≥1 year, and fluent in French) and were representative of the target population. The session, lasting ~60–90 min, was facilitated by trained bilingual moderators using a semi‐structured guide to explore participants’ understanding of the translated items, linguistic clarity, and cultural relevance. Discussions were audio‐recorded with participant consent, transcribed verbatim, and analyzed using member checking to enhance interpretive accuracy [41].

Given the prevalidation purpose of this phase, a single FGD was considered sufficient to identify major issues related to comprehension and translation. Findings from both the expert interviews and the FGD informed final refinements, ensuring that the French FAD‐GF12 achieved conceptual equivalence and cultural relevance prior to field validation.

### 2.5. Field Validation Sample and Participants

#### 2.5.1. Eligibility Criteria

Participants were eligible if they were aged ≥40 years, diagnosed with T2D for at least 1 year, and fluent in French. Individuals with cognitive impairment or severe comorbidities were excluded.

### 2.6. Sampling and Sample Size Justification

Participants were recruited using stratified random sampling across six health centers.

Sample size estimation was based on detecting an association between family functioning and glycemic control using regression analysis, assuming an effect size corresponding to an odds ratio of ~1.8–2.0, *α* = 0.05, and 80% power, following established approaches for sample size determination in regression models [42, 43]. The minimum required sample size was *n =* 444, increased to account for potential nonresponse.

The final sample (*n =* 512) exceeded this requirement and was also adequate for factor analysis. The dataset was randomly split into two subsamples (*n =* 256 each) for independent exploratory and confirmatory factor analyses, exceeding recommended minimum thresholds (≥200) for structural equation modeling [44, 45].

### 2.7. Data Collection and Measurements

Data were collected using a structured questionnaire capturing sociodemographic and clinical characteristics, including age, sex, marital status, education, duration of diabetes, family type, employment status, household size, and income. Anthropometric and biochemical measurements were obtained using standardized procedures. Weight was measured to the nearest 0.1 kg with participants in light clothing and without shoes, using a calibrated Hana digital scale (China). Height was measured to the nearest 0.1 cm using a stadiometer, and body mass index (BMI) was calculated as weight (kg) divided by height squared (m^2^). BMI categories followed WHO criteria: underweight (<18.5 kg/m^2^), normal weight (18.5–24.9), overweight (25–29.9), and obese (≥30) [46]. Waist circumference was measured with participants standing upright, and hip circumference was used to compute the waist‐to‐hip ratio. Abdominal obesity was defined as a ratio >0.90 for males and >0.85 for females [47]. Glycemic control was assessed using the HemoCue HbA1c 501 System, with HbA1c levels >7% indicating poor control [48].

### 2.8. Test–Retest Reliability

Test–retest reliability was assessed in a randomly selected subsample (~10%–15% of participants). The FAD‐GF12 was readministered after a 2‐week interval, consistent with standard reliability assessment practices [49]. Reliability was evaluated using intraclass correlation coefficients (ICCs), with values ≥0.70 indicating acceptable stability [49].

### 2.9. Data Analysis

Qualitative data from pretests and FGDs were analyzed using MAXQDA 2020 (VERBI Software). Audio recordings were transcribed verbatim, coded, and thematically analyzed to identify patterns related to clarity, cultural relevance, and item interpretation.

Quantitative analyses were conducted using Stata 18, with statistical significance set at *α* = 0.05. Descriptive statistics (frequencies, means, and standard deviations) were computed for sociodemographic and clinical variables. For the FAD‐GF12, negatively worded items were reverse‐coded, and mean scores <2 were interpreted as indicating healthy family functioning.

We examined the construct validity of the scale through a sequential analytic framework integrating exploratory factor analysis (EFA) and confirmatory factor analysis (CFA). EFA was conducted on the first subsample (*n* = 256) to examine the underlying factor structure of the French FAD‐GF12. Principal component analysis (PCA) was used as a data reduction approach to identify latent dimensions within the scale [44], followed by orthogonal varimax rotation to facilitate interpretability. Factor retention was guided by multiple criteria, including eigenvalues greater than 1.0, inspection of the scree plot, and the conceptual interpretability of the resulting factor solution [50]. The adequacy of the data for factor analysis was evaluated using the Kaiser–Meyer–Olkin (KMO) measure of sampling adequacy and Bartlett’s test of sphericity.

CFA was conducted on the second subsample (*n* = 256) to evaluate the factor structure identified in the exploratory analysis. Model fit was assessed using multiple complementary indices, including the root mean square error of approximation (RMSEA), standardized root mean square residual (SRMR), comparative fit index (CFI), and Tucker–Lewis index (TLI), with acceptable thresholds defined as RMSEA = 0.05–0.08, SRMR < 0.08, and CFI and TLI ≥ 0.90 [45, 51–53]. The correlation between latent factors was estimated to assess whether the dimensions reflected related but distinct constructs. Modification indices were examined to identify potential areas of model misfit, and any adjustments were implemented only when theoretically justified.

Finally, to examine the relationship between family functioning and glycemic control, we conducted both continuous and categorical analyses. Linear regression models were used to assess the association between continuous FAD‐GF12 scores and continuous HbA1c levels. Logistic regression models were used with dichotomized variables (FAD‐GF12 <2 vs. ≥2; HbA1c ≤7% vs. >7%) to facilitate clinical interpretation [54–56]. This combined approach aligns with recommendations to retain continuous analyses while allowing clinically meaningful interpretation. All models were adjusted for age, sex, education, diabetes duration, employment status, marital status, BMI, and healthcare center. Model assumptions were verified using variance inflation factors and goodness‐of‐fit tests.

### 2.10. Bias and Quality Control

Trained bilingual interviewers administered standardized questionnaires. Selection bias was minimized through stratified random sampling. Measurement bias was reduced using calibrated instruments and standardized protocols. Missing data were minimal (<1%) and handled using complete case analysis.

## 3. Results

### 3.1. Translation and Pretesting

Pretesting and content validation confirmed that the French version of the FAD‐GF12 was clear, culturally appropriate, and easily understood. Feedback from eight bilingual experts and participants led to minor linguistic refinements to improve clarity and contextual relevance. For example, “crisis” was replaced with “problem or difficulty” (item 2), and “bad feelings” with “tensions” (item 7). Item 12 was also rephrased to enhance conceptual equivalence. The final translated version is presented in Table 1.

**Table 1:** The French translation items and the corresponding FAD‐GF12 items.

Number	English version	French version
1	Planning family activities is difficult because we misunderstand each other	Il est difficile de planifier des activités en famille parce que nous nous comprenons mal
2	In times of crisis we can turn to each other for support	En cas de problème ou de difficulté, nous pouvons compter les uns sur les autres pour nous soutenir
3	We cannot talk to each other about the sadness we feel	Nous ne pouvons pas parler entre nous de la tristesse que nous ressentons
4	Individuals are accepted for what they are	Chaque membre de la famille est accepté tel qu’il est
5	We avoid discussing our fears and concerns	Nous évitons de parler de nos peurs et de nos inquiétudes
6	We can express feelings to each other	Nous pouvons exprimer nos sentiments les uns aux autres
7	There are lots of bad feelings in our family	Il y a beaucoup de tension dans la famille
8	We feel accepted for what we are	Nous nous sentons acceptés tels que nous sommes
9	Making decisions is a problem for our family	Prendre des décisions est difficile dans notre famille
10	We are able to make decisions about how to solve problems	Nous sommes capables de décider ensemble comment résoudre nos problèmes
11	We don’t get along well together	Nous ne nous entendons pas bien entre nous
12	We confide in each other	Nous partageons facilement nos problèmes les uns avec les autres

### 3.2. Participant Characteristics

A total of 512 adults with T2D were included in the analysis. The mean age was 52.2 ± 7.8 years, with most participants (80.9%) aged between 45 and 60 years. The majority were female (60.9%) and married (75.2%), and ~60% had completed secondary or university education. Most participants lived in nuclear families (73.0%), had low monthly incomes (<$166; 59.6%), and were self‐employed (61.7%). Clinically, the mean HbA1c level was 8.36 ± 2.11%, and only 30.5% of participants had controlled glycemic levels (HbA1c ≤7%). The mean BMI was 29.11 ± 6.30 kg/m^2^, with a high prevalence of overweight and obesity (73.8%). Detailed characteristics are presented in Table 2.

**Table 2:** Socio‐demographic and clinical characteristics of pilot test participants (*n* = 512).

Variables	Frequency *n* (%)
Age, mean ± SD (years)	52.20 ± 7.81
<45	52 (10.16)
45–60	414 (80.86)
>60	46 (8.98)
Gender	
Female	312 (60.94)
Male	200 (39.06)
Marital status	
Married	385 (75.20)
Single	64 (12.50)
Widow/widower	63 (12.30)
Education	
No formal education	89 (17.38)
Primary	115 (22.46)
Secondary	201 (39.26)
University	107 (20.90)
Diabetes duration, mean ± SD (years)	7.88 ± 6.85
Type of family	
Monogamy	374 (73.05)
Polygamy	138 (26.95)
Family size	
≤5	344 (67.19)
>5	168 (32.81)
BMI, mean ± SD, *n* = 510 (kg/m^2^)	29.11 ± 6.30
Underweight	11 (2.17)
Normal	122 (24.06)
Overweight	177 (34.91)
Obese	197 (38.86)
Waist/hip ratio, mean ± SD (cm)	0.89 ± 0.10
Low	209 (40.98)
Moderate	105 (20.59)
High	196 (38.43)
Hb A1c (mean ± SD)	8.36 ± 2.11
≤7%	156 (30.47)
>7%	356 (69.53)
Monthly income	
<$166	305 (59.57)
$166‐$332	134 (26.17)
>$332	73 (14.26)

Abbreviation: SD, *s*tandard deviation.

### 3.3. EFA

EFA was conducted on the first subsample (*n =* 256). The KMO measure indicated very good sampling adequacy (KMO = 0.88), and Bartlett’s test of sphericity was statistically significant (*p*  < 0.001), confirming that the data were suitable for factor analysis.

Two factors were retained based on eigenvalues greater than 1, scree plot inspection, and conceptual interpretability. Factor 1 comprised positively worded items (items 2, 4, 6, 8, 10, and 12), representing supportive and adaptive aspects of family functioning. Factor 2 included negatively worded items (items 1, 3, 5, 7, 9, and 11), reflecting conflict and dysfunction within the family. Factor loadings ranged from 0.658 to 0.951, indicating strong item‐factor relationships. Together, the two factors explained 64% of the total variance, supporting a robust two‐dimensional structure of the scale (Table 3). The Cronbach’s alpha for the French General Functioning 12‐item scale (GF12) was 0.85, signifying good reliability.

**Table 3:** Results from exploratory factor analysis (EFA) of the French GF12 (*n* = 256).

Item (Statement)	Pattern matrix	Factors	
		1	2
8	We feel accepted for what we are	0.951	—
10	We are able to make decisions about how to solve problems	0.947	—
12	We confide in each other	0.911	—
2	In times of crisis, we can turn to each other for support	0.903	—
6	We can express feelings to each other	0.888	—
4	Individuals are accepted for what they are	0.672	—
5	We avoid discussing our fears and concerns	—	0.918
7	There are lots of bad feelings in our family	—	0.848
1	Planning family activities is difficult because we misunderstand each other	—	0.773
3	We cannot talk to each other about the sadness we feel	—	0.747
9	Making decisions is a problem for our family	—	0.675
11	We don’t get along well together	—	0.658

### 3.4. CFA

CFA was conducted on the second subsample (*n =* 256) to evaluate the factor structure identified in the EFA. Model fit indices indicated good fit to the data: CFI = 0.94, TLI = 0.92, RMSEA = 0.06, and SRMR = 0.04, all within recommended thresholds.

**Figure 1:**
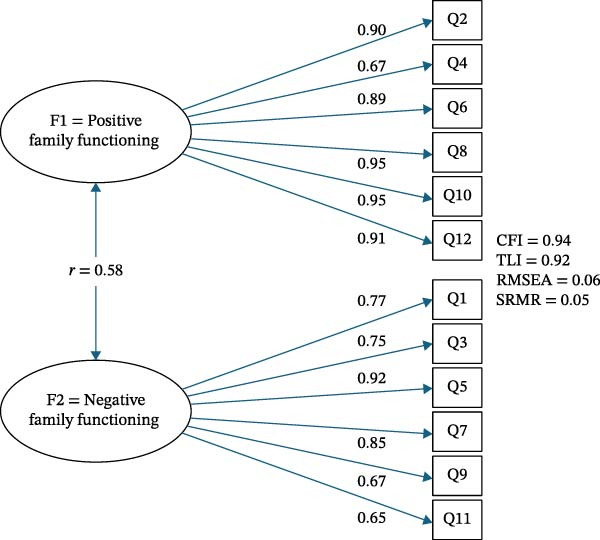
Confirmatory factor analysis (CFA) model of the French FAD‐GF12.

The correlation between the two latent factors was moderate (*r* = 0.58), indicating that the dimensions are related but represent distinct aspects of family functioning. This finding supports the bidimensional structure of the FAD‐GF12, distinguishing between positive and negative family functioning. Modification indices were examined to assess potential areas of model misfit; however, no theoretically justified modifications were identified, and the original model was retained (Figure 1).

### 3.5. Association Between Family Functioning and Glycemic Control

Overall, 56.8% of participants reported healthy family functioning (FAD‐GF12 < 2).

In linear regression models, better family functioning was significantly associated with lower HbA1c levels (*β* = −0.34, SE = 0.12, *p* = 0.003), indicating that healthier family environments are associated with improved glycemic outcomes.

Consistent findings were observed in logistic regression analyses. Participants with healthy family functioning had significantly higher odds of achieving good glycemic control (HbA1c ≤7%) compared to those with unhealthy family functioning (AOR = 2.04; 95% CI: 1.27–3.27; *p* = 0.004), after adjusting for age, sex, education, BMI, diabetes duration, marital status, employment status, and health center. Crude and adjusted estimates were similar in direction and magnitude, supporting the robustness of the association (Table 4).

**Table 4:** Association between family functioning and glycemic control^a^.

Variable	Linear regression (HbA1c, continuous)		Logistic regression (HbA1c ≤7%)	
	*β* (SE)	*p*‐Value	AOR (95% CI)	*p*‐Value
FAD‐GF12 score	−0.35 (0.12)	0.003	2.04 (1.27–3.27)	0.004

Abbreviations: AOR = adjusted odds ratio; CI = confidence interval.

^a^95% CI = 95% confidence interval. Independent variable = healthy family functioning. *Y*, dependent variable = glycemic control (HbA1c). Linear regression models examined the association between continuous FAD‐GF12 scores and HbA1c levels. Logistic regression models examined the likelihood of achieving good glycemic control (HbA1c ≤7%). All models were adjusted for age, sex, education, BMI, diabetes duration, and health center.

## 4. Discussion

This study aimed to translate, culturally adapt, and psychometrically validate the French version of the McMaster Family Assessment Device—General Functioning Subscale (FAD‐GF12) among adults with T2D in Benin. Overall, the findings demonstrate that the French FAD‐GF12 is a reliable, valid, and culturally appropriate instrument for assessing family functioning in a Francophone African context.

The scale exhibited strong psychometric properties, including good internal consistency (Cronbach’s *α* = 0.85) and satisfactory test–retest reliability (ICC > 0.75), indicating both internal coherence and temporal stability. These findings are consistent with prior validations conducted in diverse populations, including Portuguese, Spanish, Dutch, and Malaysian samples [28, 32–35]. Together, these results support the robustness and cross‐cultural applicability of the instrument.

Exploratory and confirmatory factor analyses supported a two‐factor structure distinguishing positively and negatively worded items, consistent with previous studies [32–35]. The model demonstrated good fit (CFI = 0.94, TLI = 0.92, RMSEA = 0.06, SRMR = 0.04) and explained 64% of the total variance. Importantly, the moderate correlation between the two latent factors (*r* = 0.58) indicates that the dimensions are related but conceptually distinct. This finding supports a bidimensional representation of family functioning, capturing both supportive (e.g., communication, emotional support) and dysfunctional (e.g., conflict, avoidance) relational processes. The separation of positive and negative dimensions is consistent with prior psychometric evidence and reflects meaningful variation in family dynamics rather than redundancy.

From a theoretical perspective, the McMaster Model of Family Functioning aligns well with collectivist family systems commonly observed in African settings. These systems are characterized by interdependence, shared responsibilities, and collective decision‐making [9–11, 19–21]. The distinction between positive and negative family processes observed in this study reflects these relational dynamics and underscores the relevance of the FAD‐GF12 for capturing culturally meaningful aspects of family functioning. By validating the scale in a Francophone African population—where few instruments have been rigorously adapted—this study extends existing evidence and addresses an important methodological gap.

The association between family functioning and glycemic control further supports the construct validity of the scale and highlights the clinical relevance of family dynamics. In linear regression analyses, better family functioning was significantly associated with lower HbA1c levels, indicating that supportive family environments contribute to improved metabolic outcomes. These findings were corroborated by logistic regression analyses, which showed that participants with healthier family functioning had significantly higher odds of achieving good glycemic control. The consistency between continuous and categorical analyses strengthens the robustness of these findings and aligns with methodological recommendations to retain continuous measures while allowing clinically meaningful interpretation [54–56].

These findings are consistent with a growing body of literature demonstrating that family support plays a critical role in diabetes self‐management, treatment adherence, and psychological well‐being [12–14, 29–31]. Supportive family environments can enhance motivation, self‐efficacy, and adherence behaviors, while effective communication and emotional support may buffer stress and its physiological effects on glycemic regulation [29–31]. Conversely, dysfunctional family dynamics may increase psychological distress, reduce adherence, and contribute to poorer metabolic outcomes [12, 13, 57]. In African contexts, where family networks often extend beyond the nuclear household, these influences may be even more pronounced [9–11].

The validated French FAD‐GF12 provides healthcare providers with a practical, reliable, and culturally appropriate tool for assessing family functioning in resource‐constrained settings. Its brevity and ease of administration make it suitable for integration into outpatient and community‐based diabetes care. Importantly, the bidimensional structure enhances clinical interpretation by capturing both supportive and dysfunctional aspects of family relationships. Incorporating family functioning assessments into routine care may facilitate the development of targeted, family‐centered interventions that improve adherence and glycemic outcomes [58–60]. This approach is particularly relevant in low‐ and middle‐income settings, where family systems serve as a primary source of psychosocial and practical support.

## 5. Strengths and Limitations

This study has several notable strengths. It employed a rigorous mixed‐methods design combining expert review, participant feedback, and quantitative validation in a large sample (*n* = 512). The use of split‐sample validation (EFA and CFA) enhances the robustness of the findings and reduces the risk of overfitting [45, 51, 52]. Additionally, the integration of qualitative pretesting ensured cultural relevance and conceptual equivalence of the translated instrument [36–38]. To our knowledge, this is one of the first studies to validate a family functioning instrument in a Francophone African population.

However, several limitations should be considered. First, the cross‐sectional design limits causal inference regarding the relationship between family functioning and glycemic control. Second, reliance on self‐reported measures may introduce recall and social desirability bias. Third, participants were recruited from healthcare facilities, which may limit generalizability to individuals not engaged in care. Finally, although the two‐factor structure demonstrated good fit, the separation of positive and negative items may partly reflect method effects related to item wording. Future research should explore alternative modeling approaches, such as bifactor or method‐factor models, to further examine the dimensionality of the scale [45, 51, 52]. Longitudinal studies are also needed to assess predictive validity and causal pathways.

## 6. Conclusion

In conclusion, the French FAD‐GF12 is a psychometrically robust and culturally relevant instrument for assessing family functioning among French‐speaking adults with T2D in Benin. Its strong reliability, valid factor structure, and demonstrated association with glycemic control support its use in both research and clinical practice. Integrating family functioning assessments into diabetes care may facilitate the development of culturally tailored, family‐centered interventions that improve adherence, psychosocial well‐being, and metabolic outcomes. Future research should further explore how family dynamics interact with behavioral, psychological, and socioeconomic factors to influence long‐term diabetes outcomes across diverse African settings.

## Author Contributions

Halimatou Alaofè led study conception and design, with input from John Ehiri and Waliou Amoussa Hounkpatin. Halimatou Alaofè conducted data collection with the assistance of Waliou Amoussa Hounkpatin and Lionel Medenou. Halimatou Alaofè, John Ehiri, and Abidemi Okechukwu performed data analysis and interpretation of results. Halimatou Alaofè and Raymond Yurika conceived of the manuscript together and participated in planning the writing, workflow, and timeline. Halimatou Alaofè wrote the first draft. Abidemi Okechukwu, Waliou Amoussa Hounkpatin, Lionel Medenou, andJohn Ehiri reviewed the first draft and offered substantial revisions, which Halimatou Alaofè and Raymond Yurika iteratively incorporated into second and third drafts.

## Funding

This work was supported by the National Institutes of Health Fogarty International Center (Grant K01TW012422).

## Disclosure

All authors read and approved of the final manuscript.

## Ethics Statement

The study protocol received initial approval from the institutional review boards of the University of Arizona and the National Ethics Committee for Health Research (CNRES) in Benin. Then, permission was obtained from the participating centers to proceed with the study. Strict confidentiality was maintained throughout, and the study adhered to Helsinki guidelines to ensure participants’ anonymity. This study adhered to the STROBE (Strengthening the Reporting of Observational Studies in Epidemiology) guidelines for cross‐sectional studies.

## Consent

Participants were asked to complete written consent forms after study objectives were explained. Consent was obtained from those willing to participate in the various phases of the study.

## Conflicts of Interest

The authors declare no conflicts of interest.

## Data Availability

The datasets used and/or analyzed during the current study are available from the corresponding author upon reasonable request.
